# Intracranial Hemorrhage as the Primary Presentation of Metastatic Ewing Sarcoma: A Case Report

**DOI:** 10.7759/cureus.106665

**Published:** 2026-04-08

**Authors:** Alex Cha, Mark Krel

**Affiliations:** 1 Department of Biomedical Education, California Health Sciences University College of Osteopathic Medicine, Clovis, USA; 2 Department of Neurosurgery, Community Neurosciences Institute, Fresno, USA; 3 Department of Specialty Medicine, California Health Sciences University College of Osteopathic Medicine, Clovis, USA

**Keywords:** brain metastasis, ewing sarcoma, ewing sarcoma metastasis, neuro-oncology, pediatric oncology, primary bone tumors, soft tissue sarcoma

## Abstract

Ewing sarcoma (ES) is an aggressive malignant neoplasm that most commonly arises from bone or soft tissue in children and young adults. Although metastatic disease is frequently encountered, intracranial involvement is rare and often presents with nonspecific neurologic symptoms, creating diagnostic and management challenges. We report the case of a young adult with a known history of ES who presented with progressive neurologic decline. Neuroimaging revealed a large intracranial mass associated with extensive vasogenic edema and midline shift, prompting urgent neurosurgical evaluation. The patient underwent surgical intervention for decompression and diagnostic clarification, followed by multidisciplinary oncologic management. This case emphasizes the importance of maintaining a high index of suspicion for metastatic disease in patients with a history of ES who develop new neurologic symptoms. It also highlights the role of early imaging, timely neurosurgical involvement, and coordinated care in addressing rare intracranial manifestations of systemic malignancy. Recognition of this uncommon presentation is essential to guide appropriate management and improve clinical outcomes.

## Introduction

Ewing sarcoma (ES) is a rare, aggressive bone and soft tissue malignancy that most commonly affects adolescents and young adults. The hallmark chromosomal translocation, t(11;22)(q24;q12), creates the ES RNA-binding protein 1/Friend leukemia integration 1 (*EWSR1-FLI1*) fusion gene, which is pivotal in tumor pathogenesis [1]. ES frequently presents in the long bones, pelvis, or ribs, with metastasis typically involving the lungs and bones [2]. Brain metastasis is extremely rare, underscoring the need to consider central nervous system (CNS) involvement even in unusual presentations. Symptoms of brain metastasis may include headaches, nausea, seizures, cognitive changes, and focal neurologic deficits [3]. Timely CNS monitoring can improve outcomes in ES patients, particularly when brain metastasis is detected and treated promptly [4].

Sarcomas are neoplasms of bone and soft tissue derived from a mesenchymal origin. Ewing sarcoma of the bone (ETB) is one of three neoplasms categorized under the Ewing sarcoma family of tumors (EFTs), with the other two being extraosseous Ewing tumor (EOE) and peripheral primitive neuroectodermal tumor (PPNET). ETB is the most common subtype and typically arises in the diaphysis of long bones, axial skeleton, and flat bones such as the pelvis or scapula [2].

The *EWSR1-FLI1* fusion protein is the key driver of ES tumorigenesis. The *EWSR1* gene encodes an RNA-binding protein involved in transcription and splicing, whereas the *FLI1* gene encodes an ETS-family transcription factor that plays an important role in angiogenesis and hematopoiesis. Overactivation of *FLI1* can lead to repression of the retinoblastoma protein (RB) encoded by the *RB1* gene and increased expression of B-cell lymphoma 2 (BCL-2), encoded by the *BCL2 *gene, resulting in enhanced cell survival and proliferation [5].

ES arises due to a chromosomal translocation between chromosomes 22 and 11. This rearrangement produces an in-frame fusion between the amino terminus of *EWSR1* and the carboxyl terminus of *FLI1*, corresponding to t(11;22)(q24;q12). The resulting *EWSR1-FLI1* fusion protein functions as an aberrant transcription factor by combining the strong transactivation domain of *EWSR1* with the ETS DNA-binding domain of *FLI1* [6,7]. This chimeric protein promotes oncogenic transcriptional programs that drive tumorigenesis and the development of ES. Reverse transcription polymerase chain reaction (RT-PCR) is commonly used to detect the fusion transcript and confirm this gene rearrangement for diagnostic purposes.

ES is a rare, highly aggressive malignancy predominantly affecting adolescents and young adults, with a higher incidence in Caucasians compared with African Americans [8]. It primarily involves bone and soft tissue, while extraosseous involvement is less common. The lungs and bones remain the most frequent sites of metastasis, whereas brain metastasis is exceedingly rare [9]. The development of intracranial disease presents significant challenges for clinical management and prognosis.

ES demonstrates a male predominance, with an approximate ratio of 1.5:1. Diagnosis typically occurs around 14-15 years of age, and delayed diagnosis correlates with worse outcomes. A study of 4,600 patients reported a five-year survival rate of 61.8% for those under 40 years of age compared with 44.6% for those over 40 years [10].

In children, ES most commonly arises in bone, whereas in adults, more than half of cases involve soft tissue. Although systemic spread frequently involves the lungs and bones, brain metastasis remains rare. Hepatoma-derived growth factor has been implicated in promoting metastasis by regulating cell-matrix adhesion and migration in ES cells [7]. A cohort study reported one-, two-, and five-year survival rates of 89.08%, 78.08%, and 62.47%, respectively, with an overall mortality rate of 0.05 per 100,000 individuals in the United States [11].

Building on this background, we present the details of the current case to further explore the clinical and diagnostic implications of brain metastasis in ES.

## Case presentation

A 25-year-old male was initially diagnosed with primary ES of the T11-T12 vertebral region on June 12, 2023, and underwent nine rounds of multimodal treatment with chemotherapy and radiation. Intracranial involvement was identified approximately one year following the initial diagnosis, representing a metachronous recurrence. At the time of presentation, staging did not demonstrate evidence of widespread systemic disease, and the lesion was determined to be a solitary intracranial metastasis.

During admission, he reported new-onset, persistent headaches, intermittently relieved by hydrocodone-acetaminophen. Given his underlying malignancy, this symptom was considered concerning for intracranial involvement despite the absence of focal neurologic deficits on initial examination, prompting further evaluation. Admission laboratory studies demonstrated an elevated D-dimer level and thrombocytopenia, as summarized in Table 1. The elevated D-dimer and thrombocytopenia were interpreted in the context of underlying malignancy, raising concern for a prothrombotic and consumptive coagulopathic state, which may predispose to both hemorrhagic and thrombotic intracranial events.

**Table 1:** Hospital admission laboratory values

Test	Value	Units	Reference Range
D-dimer	1.87	µg/mL	<0.50
Platelet	92	×10³/µL	150–450

Day 1 (initial imaging and consultation)

**Figure 1:**
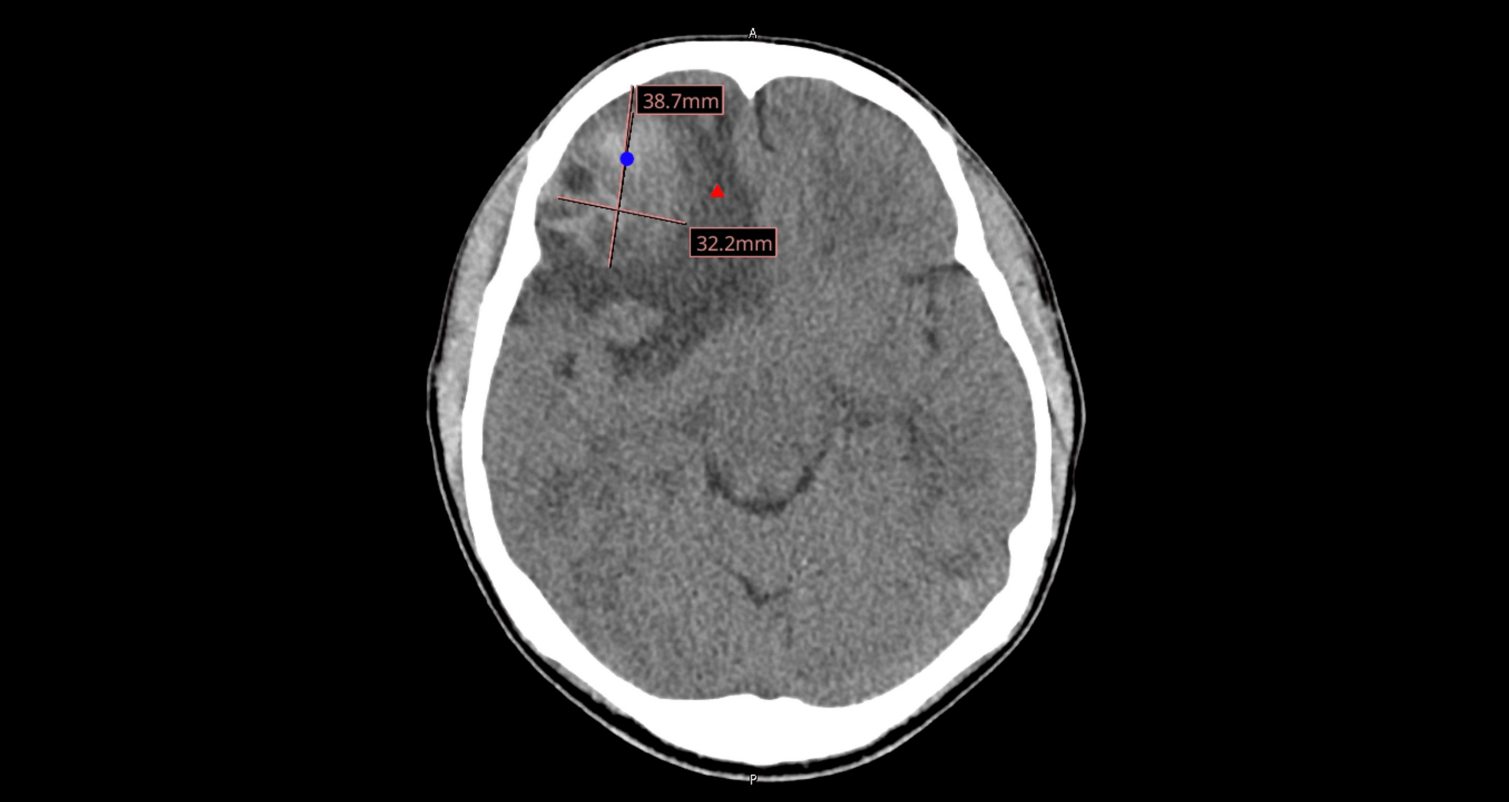
CT scan showing right frontal hemorrhage (blue circle) with vasogenic edema (red triangle) and midline shift.

A non-contrast computed tomography (CT) scan of the head was ordered, which revealed an acute right frontal intraparenchymal hemorrhage measuring 3.9 cm × 3.2 cm, with marked surrounding vasogenic edema resulting in a 4-mm leftward midline shift and subfalcine herniation (Figure 1). This prompted an urgent referral to neurosurgery.

Neurosurgical Evaluation and Imaging

During neurosurgical consultation, the patient denied vision changes, dizziness, nausea, vomiting, bowel or bladder dysfunction, or extremity weakness or numbness. Neurologic examination was nonfocal, with intact cranial nerves II-XII and normal motor and sensory function in all extremities. Pupils were 6 mm bilaterally. Despite this, the presence of new-onset headache in a patient with a known malignancy prompted urgent neuroimaging.

A magnetic resonance imaging (MRI) of the brain with and without contrast was obtained for further evaluation.

Day 2 (MRI imaging and surgical planning)

**Figure 2:**
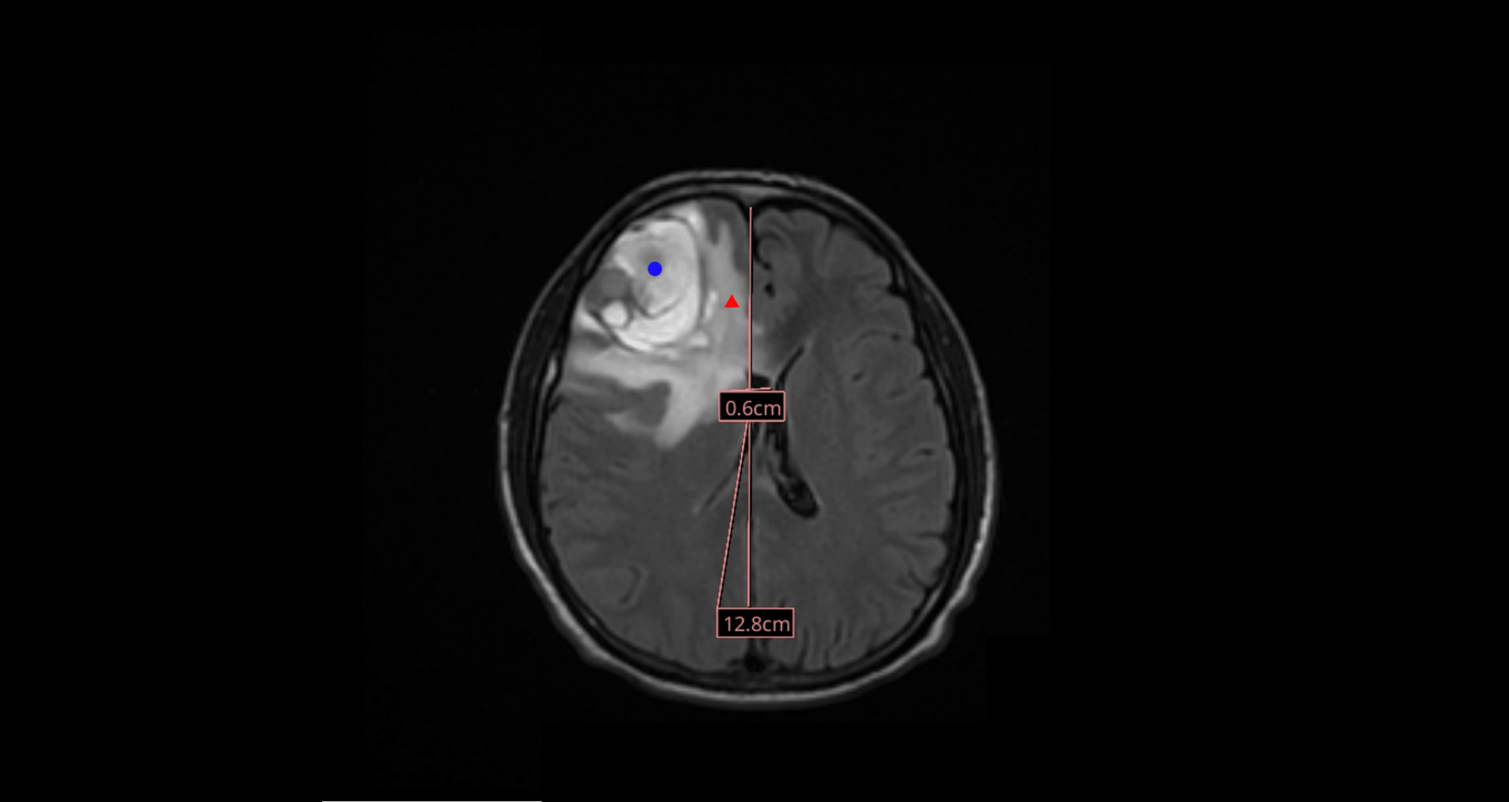
MRI (axial view) showing right frontal hemorrhage (blue circle) with edema (red triangle).

**Figure 3:**
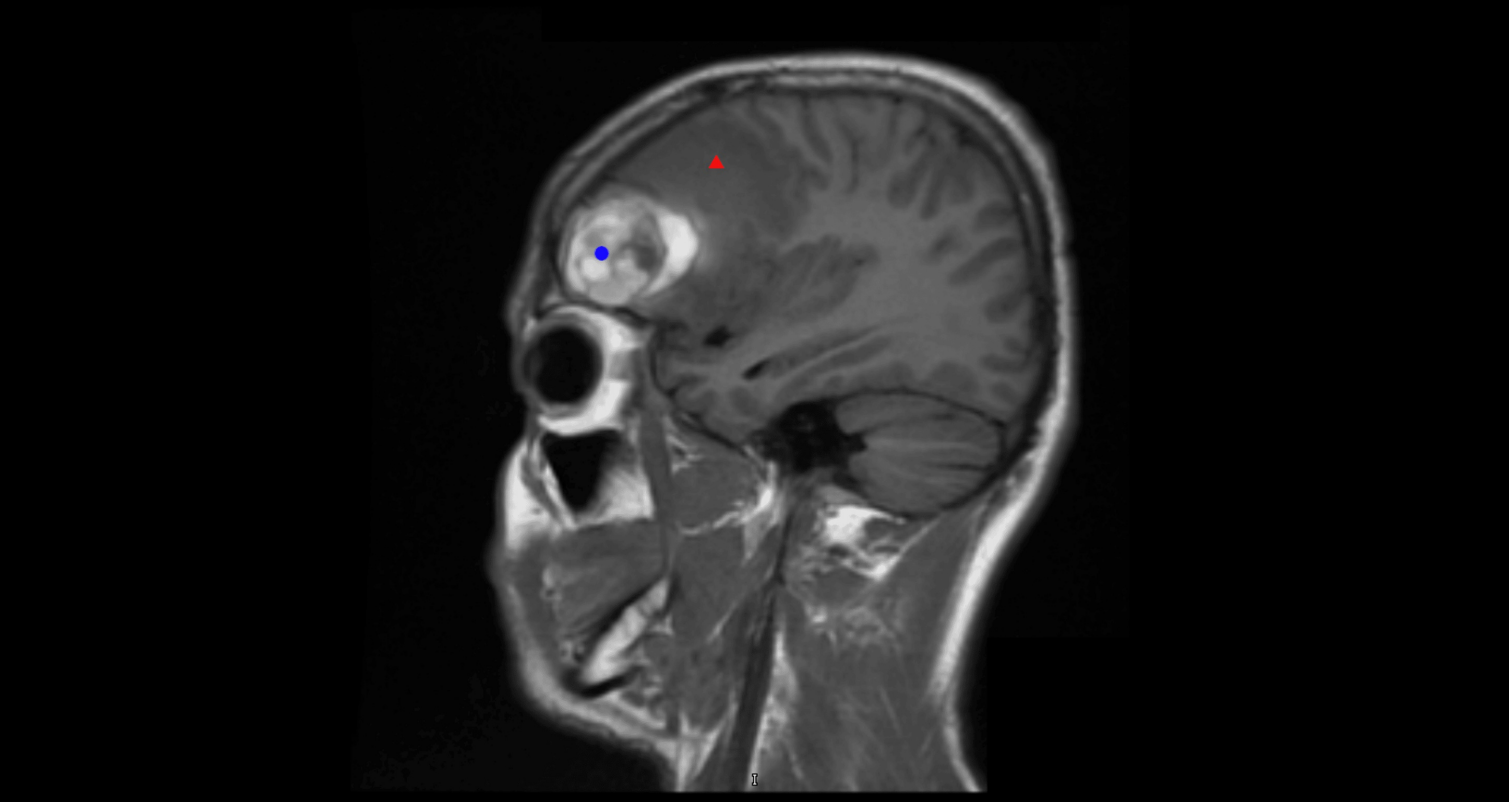
MRI (sagittal view) showing right frontal hemorrhage (blue circle) with edema (red triangle).

Multiplanar, multisequence MRI of the brain with intravenous gadoterate meglumine (10 mL) demonstrated a 4.3-cm hemorrhagic lesion in the right frontal lobe, with peripheral enhancement extending to approximately 5.4 cm. There was surrounding vasogenic edema with associated mass effect, without evidence of hydrocephalus or extra-axial collection (Figures 2, 3). In the context of known ES, these findings were highly concerning for hemorrhagic metastatic disease.

Physical examination findings remained unchanged, except for a decrease in pupil size to 4 mm bilaterally. After discussing the imaging results, the patient consented to tumor resection. No abnormalities were found on electrocardiogram (ECG), and the patient was cleared for surgery. Preoperative laboratory values are shown in Table 2.

**Table 2:** Preoperative laboratory values WBC, white blood cells; RBC, red blood cells; Hb/Hgb, hemoglobin; Hct, hematocrit; Plt, platelets

Test	Value	Units	Reference Range
WBC	4.2	×10³/µL	4.0–11.0
RBC	3.52	×10⁶/µL	4.2–5.9
Hb/Hgb	10.7	g/dL	13.5–17.5
Hct	30.6	%	41–53
Plt	105	×10³/µL	150–450

Day 3 (surgical intervention and pathological findings)

Given the presence of a hemorrhagic lesion with surrounding edema and associated mass effect, in the setting of known malignancy, there was high suspicion for metastatic disease. These imaging findings, combined with the risk of neurologic deterioration, prompted urgent neurosurgical intervention for both diagnostic confirmation and therapeutic decompression.

The patient underwent a right frontal craniotomy for tumor resection. The intraoperative specimen was sent for pathological evaluation, which demonstrated increased cellularity with an atypical lymphoid-appearing population, raising concern for a lymphoproliferative process, primary brain neoplasm, or reactive changes.

Definitive histopathological evaluation of the resected specimen revealed a small round blue cell tumor, prompting a broad differential diagnosis, including lymphoma, metastatic small cell carcinoma, and other round cell sarcomas such as ES, given the overlapping cytologic features among these entities.

Further diagnostic evaluation demonstrated strong membranous CD99 positivity, a characteristic marker associated with ES. Molecular analysis confirmed the presence of the EWSR1-FLI1 fusion, establishing the diagnosis of metastatic ES consistent with the patient’s known primary tumor.

There were no intraoperative complications.

Postoperative course

The patient was admitted to the neuro-intensive care unit for continuous monitoring. Postoperative examination revealed left-sided weakness and a spiked gaze, prompting a code stroke. Further imaging was ordered to assess for stroke or other complications.

CT Angiogram

The head and neck CT angiogram revealed no evidence of large vessel occlusion, aneurysm, or sinus thrombosis. Neurology reviewed the imaging and canceled the stroke code, determining that the weakness was likely due to surgical swelling and mass effect.

Follow-up Imaging

CTH without contrast: Postsurgical changes were noted in the right frontal lobe with mild hemorrhage, stable vasogenic edema, mass effect on the right lateral ventricle, and an unchanged right-to-left midline shift.

**Figure 4:**
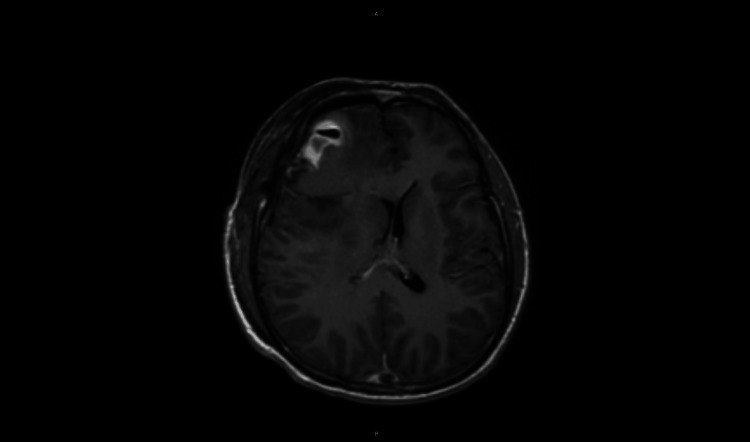
Postoperative MRI showing residual tumor and ischemic changes.

MRI of the head with and without contrast: Expected edema, fluid, and hemorrhagic products were noted in the operative cavity in the right frontal lobe, with a 0.2-cm shift of the midline. Residual tumor was noted in the region of the operative cavity. Postoperative MRI of the brain indicated new ischemic changes in the right insula and basal ganglia (Figure 4). The ischemic changes likely reflected transient perioperative alterations in regional cerebral perfusion related to vascular manipulation, local edema, and retraction during tumor resection. These findings were closely monitored clinically and radiographically, with no evidence of significant neurologic deterioration.

The patient received levetiracetam (Keppra) for seizure prophylaxis for seven days. Sodium was maintained between 140 and 145 mEq/L using hypertonic saline. Symptoms improved, and he was discharged on dexamethasone 2 mg twice daily.

Discharge

The patient was stable and discharged on dexamethasone (Decadron) 2 mg every 12 hours, with plans to continue this dosage until evaluation by the oncology outpatient team.

Emergency department return after physical altercation

The patient returned to the emergency department after a physical altercation, complaining of a severe right frontal headache (10/10). He denied vision changes, focal numbness, weakness, or speech changes. Laboratory work revealed an elevated white blood cell count of 16 × 10³/µL. A CT scan indicated worsening cerebral edema, and the patient was admitted to the neuro-medicine service. He was started on hypertonic saline 2% with sodium checks every 4 hours (target: 140-145 mEq/L), and Decadron was tapered. His headache eventually improved, and he was discharged the following day.

## Discussion

ES is an aggressive malignancy predominantly affecting adolescents and young adults. While ES commonly metastasizes to the lungs and bones, brain metastasis is exceptionally rare. The molecular hallmark of ES, the *EWSR1-FLI1* fusion gene resulting from t(11;22)(q24;q12), plays a central role in tumorigenesis [1]. This chimeric transcription factor alters gene expression involved in cellular proliferation, survival, and differentiation. It promotes oncogenic transcriptional programs that drive tumorigenesis and the development of ES. Dysregulation of downstream pathways, including suppression of tumor suppressor activity (e.g., *RB1*) and upregulation of anti-apoptotic signaling (e.g., *BCL-2*), contributes to the aggressive clinical behavior and metastatic potential of ES [5]. These molecular mechanisms may help explain the tumor’s ability to disseminate to atypical sites such as the CNS.

Here, we report a case of a 25-year-old male with brain metastasis from ES, adding to the limited literature on this unusual presentation. The patient initially presented with intermittent headaches and later developed neurologic deficits, including left-sided weakness and altered mental status. Imaging studies, notably CT and MRI, were instrumental in diagnosing the brain metastasis and guiding subsequent management.

Brain metastasis in ES often manifests with headaches, seizures, or changes in mental status. This case underscores the importance of considering brain metastasis in ES patients, even in the absence of pronounced neurologic deficits. In this instance, CT findings revealed a right frontal intraparenchymal hemorrhage with surrounding edema, and MRI confirmed the presence of an enhancing lesion, which was subsequently verified as metastatic through postoperative analysis.

Comparable cases in the literature are scarce. For instance, a case reported by Irfan et al. described a 21-year-old female with ES originating in the knee who developed a solitary brain metastasis in the right frontal lobe [12]. Like our patient, she presented with headaches after treatment with surgical resection of the primary ES tumor and was managed with chemotherapy and radiation. The metastasis was ultimately surgically resected. While both cases share similarities, key differences include the site of primary disease (T11-T12 in our patient versus the knee joint in Irfan et al.’s case), as well as gender differences.

Other case reports have documented secondary ES metastases presenting as intracranial masses [13,14]. However, while these cases involve metastases within the cranial compartment, they do not specifically impact the brain parenchyma.

Chart reviews, such as the St. Jude Children’s Research Hospital experience, have noted a 3.3% incidence of brain metastasis in ES patients [15]. While such data highlight the rarity of brain involvement in ES, they often lack detailed, individualized approaches to management. Given the limited number of reported cases of intracranial metastases in ES, there is a paucity of data comparing postoperative ischemic changes with clinical outcomes. However, in the few cases described, including those by Irfan et al. and our own, postoperative ischemic changes have been attributed to perioperative alterations in regional cerebral perfusion and tissue manipulation and were closely monitored for signs of acute neurologic deterioration. In these cases, patients remained clinically stable, suggesting no significant impact on short term prognosis while emphasizing the importance of continued postoperative surveillance [12].

This case highlights the importance of maintaining diagnostic vigilance for atypical metastatic patterns in ES, as failure to recognize CNS involvement may alter staging, delay appropriate multidisciplinary management, and worsen prognosis. Early identification through imaging is critical to guide timely intervention and optimize therapeutic outcomes.

Limitations

This report is limited by its single-case design, restricting generalizability. Additionally, variability in reporting across existing literature makes direct comparison challenging. Further studies are needed to better define risk factors, surveillance strategies, and optimal management of CNS metastasis in ES.

## Conclusions

The primary lesson from this case is the need for heightened clinical vigilance when evaluating new neurologic symptoms in patients with a known history of ES, particularly given its atypical metastatic patterns. Although ES most commonly involves the lungs and bones, intracranial involvement should remain within the differential diagnosis, especially in the setting of new-onset or persistent headache, even in the absence of focal neurologic deficits.

This case also highlights the diagnostic complexity of such presentations, as initial pathology demonstrating an atypical lymphoid-appearing population may obscure the underlying diagnosis. In this case, however, prompt surgical intervention was pursued based on concerning imaging findings, allowing for both therapeutic resection and definitive diagnosis without delay.

In this context, neuroimaging with CT and MRI plays a critical role, as findings such as hemorrhagic lesions with associated edema and mass effect should raise concern for metastatic disease and warrant urgent neurosurgical evaluation.

Furthermore, this case emphasizes the importance of integrating clinical, radiographic, and molecular findings to accurately characterize metastatic disease. The identification of EWSR1-FLI1 fusion-positive ES reinforces the role of molecular testing as the diagnostic gold standard, particularly in cases with initially indeterminate histopathology. Recognition of these atypical presentations is essential to facilitate timely multidisciplinary management and optimize clinical outcomes, while acknowledging the inherent limitations of a single case report.
